# Progressive plasmacytoid variant bladder cancer with retroperitoneal dissemination: An autopsy case report

**DOI:** 10.1002/iju5.12167

**Published:** 2020-05-28

**Authors:** Yuki Kohada, Yasuhiro Kaiho, Jun Ito, Jotaro Mikami, Go Anan, Katsutoshi Asano, Toru Yaegashi, Kazuhiro Murakami, Yasuhiro Nakamura, Makoto Sato

**Affiliations:** ^1^ Division of Urology Faculty of Medicine Tohoku Medical and Pharmaceutical University Sendai Japan; ^2^ Division of Pathology Faculty of Medicine Tohoku Medical and Pharmaceutical University Sendai Japan; ^3^ Nihon Gene Research Laboratories Inc Sendai Japan

**Keywords:** autopsy, circulating tumor cell, pembrolizumab, plasmacytoid urothelial carcinoma, urinary bladder neoplasm

## Abstract

**Introduction:**

Plasmacytoid urothelial carcinoma is a rare and aggressive variant of bladder cancer.

**Case presentation:**

A 75‐year‐old woman presented with plasmacytoid urothelial carcinoma with retroperitoneal dissemination was treated with chemotherapy. After an unsuccessful first‐line chemotherapy with gemcitabine and cisplatin, we assessed circulating tumor cells; one such cell was found to be positive for programmed death‐ligand 1. The patient received second‐line chemotherapy with pembrolizumab. However, the tumor extended to the retroperitoneal organs, and the patient eventually died. Autopsy revealed a widespread diffuse scirrhous infiltration of the carcinoma into the retroperitoneum. However, distant metastasis was not observed.

**Conclusion:**

The evaluation of circulating tumor cells and autopsy revealed a disease state of progressive plasmacytoid urothelial carcinoma treated with pembrolizumab.

**Abbreviations & Acronyms**
CA: cancer antigen
CEA: carcinoembryonic antigen
CK: cytokeratin
CT: computed tomography
CTC: circulating tumor cell
GATA3: GATA binding protein 3
GC: gemcitabine and cisplatin
HER2: human epidermal growth factor receptor type 2
LCA: leukocyte common antigen
PD‐L1: programmed cell death‐ligand 1
PUC: plasmacytoid urothelial carcinoma
TURBT: transurethral resection of the bladder tumor

**Keynote message**

An autopsy case of progressive PUC was reported. The evaluation of CTCs and autopsy revealed a progressive PUC treated with pembrolizumab.

## Introduction

PUC is a rare and aggressive variant of bladder cancer. The studies on this disease are limited, and most studies are single‐case reports or include a small cohort of patients.^1^, ^2^, ^3^, ^4^ Because of the limited information about advanced PUC, an optimal therapy has not yet to be established. Herein, we present the case of a 75‐year‐old woman with progressive PUC who received pembrolizumab therapy after the evaluation of CTCs. However, the patient eventually died, and an autopsy was performed.

## Case presentation

**Fig. 1:**
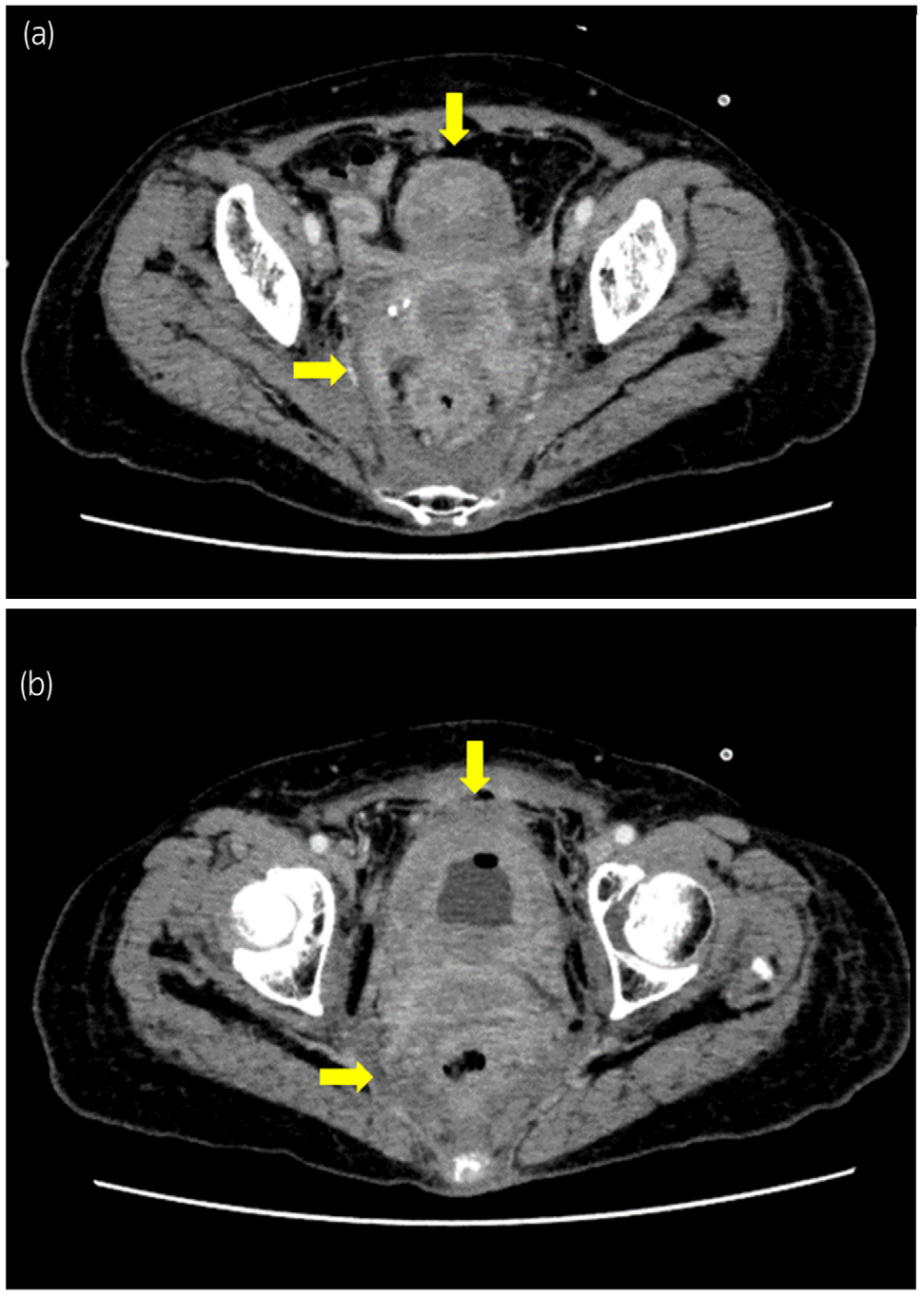
CT scan findings of the current case. (a) Ill‐defined soft tissues spread into the pelvis (arrows), which indicated retroperitoneal dissemination. (b) Diffuse thickening was observed in the bladder and rectum wall (arrows).

A 75‐year‐old woman with bilateral hydronephrosis presented to our department. Bilateral nephrostomies were performed because of acute renal failure; subsequently, renal function returned to a normal level. Laboratory examination was performed, and the following results were obtained: CEA level, 121.7 ng/mL; CA19–9 level, 2467.5 IU/mL; and CA125 level, 63.1 IU/mL. CT scan revealed diffuse thickening of the bladder and rectum wall and retroperitoneal dissemination of the tumor. However, no obvious distal and local lymphadenopathy was observed (Fig. 1). Urinary cytology revealed the presence of atypical cells. Thus, based on these findings, a preoperative diagnosis of retroperitoneal dissemination of the bladder tumor was made.

**Fig. 2:**
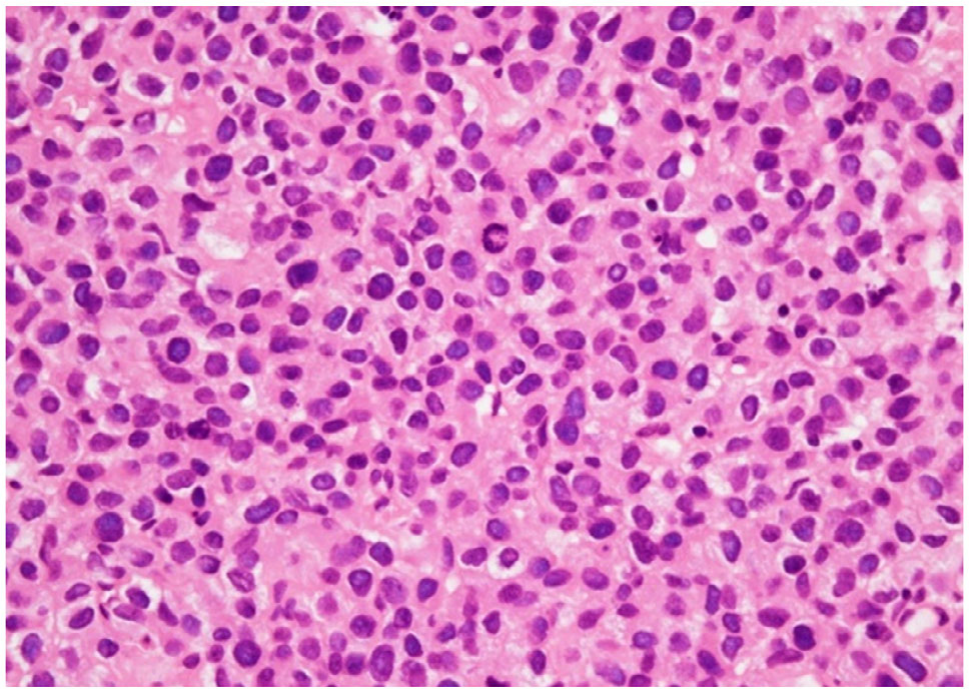
Histological findings of the current case. The tumor cells were discohesive with eccentrically located nuclei and abundant eosinophilic cytoplasm.

To diagnose the condition, TURBT was performed. The patient’s bladder capacity was extremely decreased, and the whole bladder mucosa was irregular and thick. Histopathological examination revealed that the tumor cells were discohesive with eccentrically placed nuclei and moderate to abundant eosinophilic cytoplasm, closely resembling plasma cells (Fig. 2). The tumor cells were immunohistochemically positive for CK AE/AE3, E‐cadherin, and GATA3, but negative for CK7, CK20, LCA, and PD‐L1 (data not shown). CD8‐positive T cells infiltrated the tumor. Based on these findings, the resected tumor was histologically diagnosed as PUC of the bladder.

**Fig. 3:**
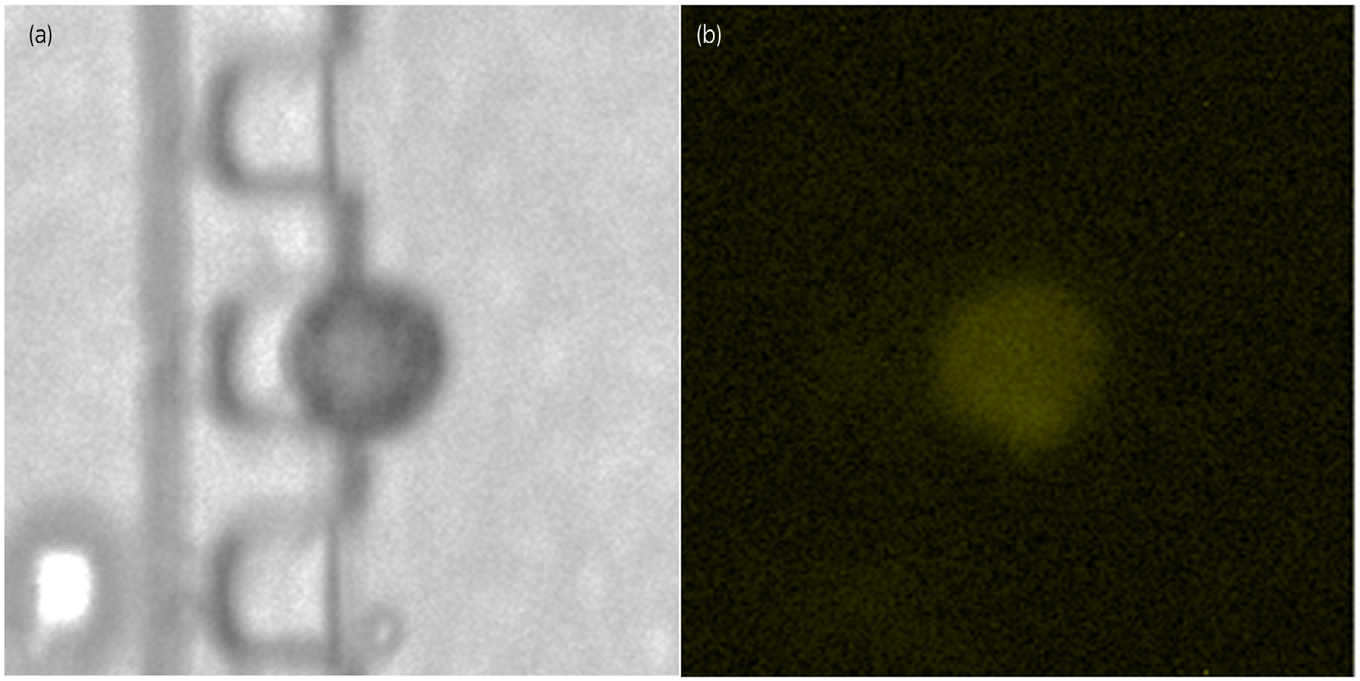
CTCs were observed in 7.5 mL of blood using the Celsee system in the current case. (a) The CTC at the bright field. (b) The CTC was immunohistochemically positive for PD‐L1 antibody.

Based on the diagnosis of cT4bN0M0 PUC of the bladder, chemotherapy with GC; gemcitabine (1000 mg/m^2^ on days 1, 8, and 15) and cisplatin (70 mg/m^2^ on day 2) were administered in two cycles. CT scan was performed after two cycles of chemotherapy and it revealed that the tumor directly reached the pancreatic head. CEA and CA19–9 levels elevated to 242.7 ng/mL and 5646.4 IU/mL, respectively. We assessed CTCs after two chemotherapy cycles, and two CTCs were found in 4 mL of blood. Although the primary tumor was negative for PD‐L1, one of the CTCs was positive for PD‐L1 (Fig. 3). Finally, 200 mg of pembrolizumab was administered as second‐line chemotherapy for urothelial carcinoma. However, the tumor extended, and the patient presented with intestinal obstruction caused by tumor invasion. The patient died 4 months after TURBT.

**Fig. 4:**
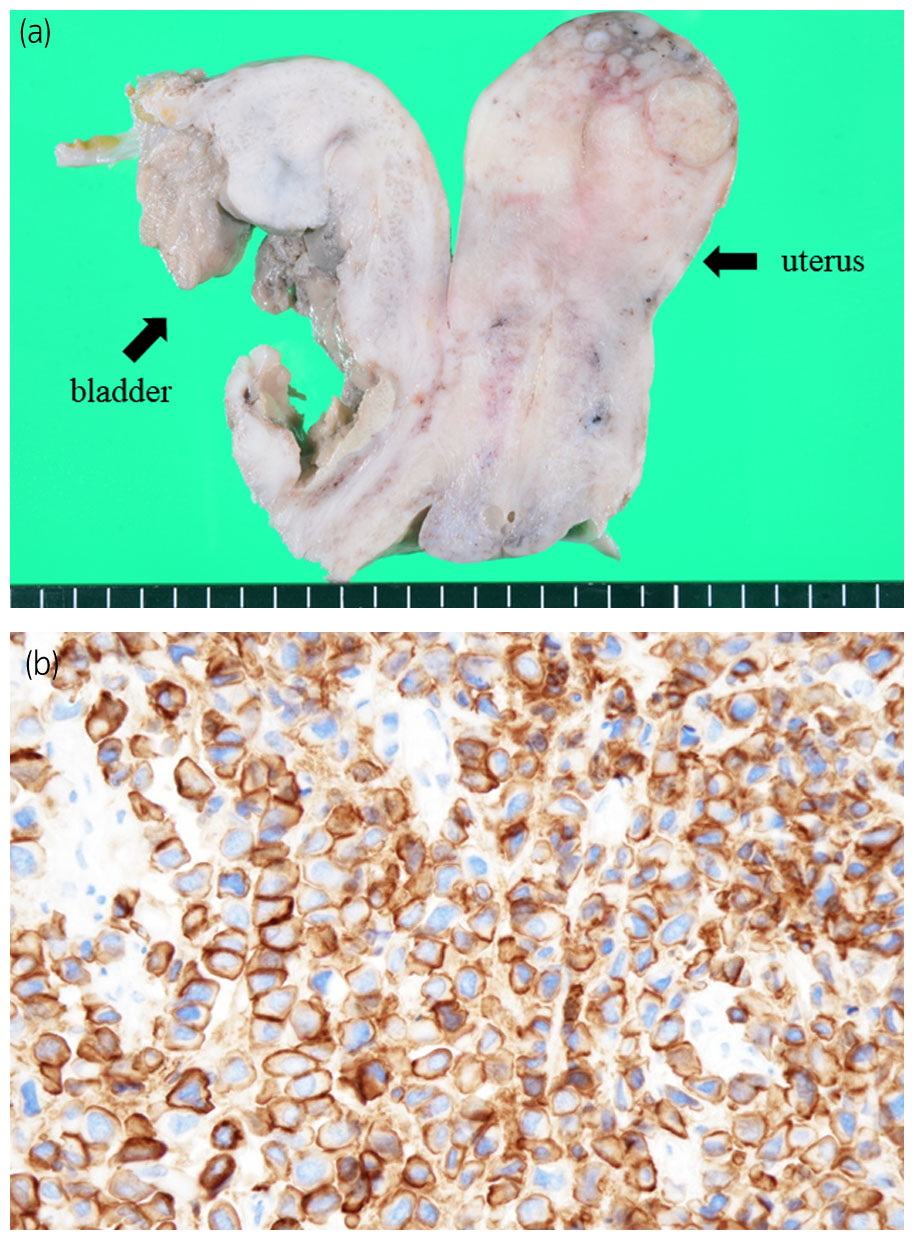
Autopsy findings of the current case. (a) The primary tumor involved the whole urinary bladder and directly invaded the anterior wall of the uterus and diffusely infiltrated into the whole myometrium of the uterus. (b) The tumor cells were immunohistologically positive for HER2.

According to the autopsy findings, the whole urinary bladder was involved by the carcinoma (Fig. 4a). Moreover, the carcinoma directly invaded the anterior wall of the uterus and diffusely infiltrated into the whole myometrium of the uterus. Autopsy revealed a widespread diffuse scirrhous infiltration of the carcinoma into the retroperitoneum that formed a membranous mass and a large tumorous mass with unclear boundary in the pancreaticoduodenal region. However, no distant metastasis was observed. Additional immunohistochemical staining revealed that the tumor cells were positive for HER2 (Fig. 4b).

## Discussion

PUC is a rare histological type of bladder tumor. Since 1991, when it was first identified,^5^ only few cases have been reported and only four case series have been performed.^1^, ^2^, ^3^, ^4^ The current case is the second autopsy report of PUC.^6^ Moreover, to the best of our knowledge, this study is the first to assess the CTCs.

The typical imaging appearance of PUC is extensive involvement of the bladder wall that frequently extends into the perivesical soft tissues.^4^ Histologically, PUC is characterized by discohesive cells with a plasmacytoid morphology and typically stains positive for GATA3 but negative for LCA and this result supports the urothelial origin of PUC.^4^, ^7^ Clinically, PUC is diagnosed at an advanced pathological stage (82%, ≥pT3), and 9% of patients present with metastasis.^4^ Generally, the treatments of local PUC are radical cystectomy and adjuvant chemotherapy, and that of advanced PUC is chemotherapy.^8^ Chemotherapy comprises methotrexate, vinblastine, doxorubicin, and cisplatin (M‐VAC), or GC is provided to almost all patients with advanced PUC. However, the chemotherapy efficiency is controversial.^9^ Although multimodality treatment including these chemotherapies is used, 57% of patients with advanced PUC survive for a short time (mean: 23 months) and the median survival time in patients with pT4 is only 15 months.^3^

We evaluated the CTCs in the peripheral blood in the current case using the Celsee system, which is an accurate and reproducible assay used to detect CTCs.^10^ In this study, we identified an active CTC in the peripheral blood, which was positive for PD‐L1. However, the primary tumor was negative for PD‐L1. In a previous study, none of the patients with PUC tested positive for PD‐L1, and pembrolizumab was not effective in all patients, except in one.^3^, ^8^, ^11^ In the current case, the tumor was negative for PD‐L1, and pembrolizumab was not clinically effective for the treatment of the primary tumor. Although active CTCs, which have potential for metastasis, were observed, distant metastasis was not observed during autopsy. This result may indicate that pembrolizumab could regulate metastasis in PUC. However, it was not effective in inhibiting the progression of the primary tumor.

A recent comprehensive molecular characterization has revealed several potential targets for UC, one of which is HER2, and their prognostic significance.^12^ Approximately 10% of patients with UC were positive for HER2.^13^ Moreover, approximately 40% of patients with the micropapillary variant, another variant of UC, present with HER2 positivity,^14^ which is associated with poor outcome.^15^ In a previous study, 80% of patients with PUC were positive for HER2.^16^ In the current case, the primary tumor was positive for HER2, which is a well‐established therapeutic target in some cancers.^17^ HER2 may be a good target for novel therapeutic strategies in the management of PUC. Further studies that assess the importance of HER2 are needed in the future.

Herein, we describe an autopsy case of progressive PUC. The evaluation of CTCs and autopsy revealed a disease state of progressive PUC treated with pembrolizumab. More data from previous reports must be obtained to validate the biological characteristics and optimal management of PUC.

## Conflict of interest

The authors declare no conflict of interest.
